# Draft genomes of multidrug-resistant *Campylobacter jejuni* and *Campylobacter coli* strains from Brazil representing novel sequence types

**DOI:** 10.1128/mra.00524-24

**Published:** 2024-09-17

**Authors:** Ana Beatriz Portes, Pedro Panzenhagen, Anamaria Mota Pereira dos Santos, Ana Carolina Silva de Jesus, Alan Clavelland Ochioni, Sheila da Silva Duque, Flávia Figueira Aburjaile, Bertram Brenig, Vasco Azevedo, Carlos Adam Conte Junior

**Affiliations:** 1 Laboratory of Advanced Analysis in Biochemistry and Molecular Biology, Department of Biochemistry, Federal University of Rio de Janeiro, Rio de Janeiro, Brazil; 2 Graduate Program in Veterinary Hygiene and Technological Processing, Faculty of Veterinary Medicine, Fluminense Federal University, Niterói, Brazil; 3 Collection of Campylobacter, Instituto Oswaldo Cruz, Fundação Oswaldo Cruz, Rio de Janeiro, Brazil; 4 Graduate Program in Food Science, Institute of Chemistry, Federal University of Rio de Janeiro, Rio de Janeiro, Brazil; 5 Department of Preventive Veterinary Medicine, Veterinary School, Federal University of Minas Gerais, Belo Horizonte, Brazil; 6 Georg August University of Göttingen, Göttingen, Germany; 7 Laboratory of Cellular and Molecular Genetics, Institute of Biological Sciences, Federal University of Minas Gerais, Belo Horizonte, Brazil; University of Maryland School of Medicine, Baltimore, Maryland, USA

**Keywords:** *Campylobacter*, multilocus sequence typing, antimicrobial resistance, antibiotic resistance, genomics

## Abstract

Whole-genome sequencing identified three previously unidentified multilocus sequence types of *Campylobacter jejuni* (ST-12332) and *Campylobacter coli* (ST-12333 and ST-12663), harboring resistance genes for multiple antimicrobial classes. The sources of isolation highlight the circulation of resistance strains within animals and humans, emphasizing the need for preventive measures.

## ANNOUNCEMENT

*Campylobacter jejuni* and *Campylobacter coli* pose significant global public health risks, causing human gastroenteritis, often linked to consumption of undercooked chicken (1). Antimicrobial treatment, particularly macrolides and fluoroquinolones, is crucial for severe infections (2). However, misuse of these drugs in veterinary settings has accelerated the emergence of antimicrobial resistance in *Campylobacter* (3). This has led to a rise in multidrug-resistant strains, increasing the risk of human infection from farm to table (4). Genome-based studies of antimicrobial resistance in *Campylobacter jejuni* and *Campylobacter coli* have recently increased (5, 6), contributing to the enrichment of data about these pathogens. In this study, we selected three phenotypically multidrug-resistant strains and identified new sequence types (STs) not previously reported, advancing genomic research on *Campylobacter* in Brazil.

The *Campylobacter* collection of the Zoonosis Laboratory—LABZOO from Oswaldo Cruz Foundation—FIOCRUZ (http://ccamp.fiocruz.br/) provided the strains for this study, where they were stored in 20% of glycerol at −80°C until use. The strains were isolated and identified following the *Campylobacter* technical manual of laboratory diagnosis (7). Briefly, the samples were suspended in 0.1% peptone water and inoculated onto selective Columbia agar supplemented with activated charcoal and antibiotic solution; after incubation of the plates at 42°C in a jar of microaerophilic environment for 48 hours, gray-colored, transparent, and glossy colonies were selected, and a Gram-staining examination was performed. Colonies with Gram-negative, gull-wing, and spiral-shaped bacteria were selected for antibiotic sensitivity and biochemical tests to differentiate *Campylobacter* species (7). We received the three strains isolated on plates, already phenotypically characterized by the Collection of *Campylobacter* (CCAMP). Three colonies were selected from the plates for DNA extraction using the QIAGEN DNeasy Blood and Tissue kit (Qiagen, Gaithersburg, MD, USA), following the manufacturer’s instructions. The DNA library was prepared with the Nextera XT DNA sample preparation kit (Illumina Inc., San Diego, CA, USA) following a 2 × 150 bp paired-end library protocol compatible with the HiSeq 2500 platform (Illumina Inc., San Diego, CA, USA). The quality analysis of the raw reads was performed with FastQC v.0.11.9 (8). Low-quality regions and adapters were trimmed with Fastp software (9). Unicycler v.5.0.0 (10) software was utilized to assemble sequences through SPAdes v. 3.14.0 (11). Taxonomic confirmation was performed by KmerFinder v. 3.0.2 (12). AMR genes and point mutations encoding resistance to beta-lactams (*bla*_OXA-193_), quinolones (*gyrA*_T86I), macrolides (50S_L22_A103V and 23S_A2075G), tetracycline (*tet*(O)), and heavy metals (*acr3* and *arsP*) were detected in the three sequences through AMRFinderPlus v3.11.14 (13). Furthermore, the STs were determined by submitting the sequences to PubMLST (14). The seven housekeeping genes in *Campylobacter* are *aspA*, *glnA*, *gltA*, *glyA*, *pgm*, *tkt,* and *uncA* (15). In this study, we identified three new combination loci of these genes (Table 1). Standard default parameters were utilized for all bioinformatics software analyses. Raw reads were uploaded to the National Center for Biotechnology Information (NCBI) (Bethesda, MD, USA), and contigs were annotated using the NCBI Prokaryotic Genome Annotation Pipeline (PGAP) v6.5 (16). Genome assembly features, resistance genes, and epidemiological data are summarized in Table 1.

**TABLE 1:** Genome assembly features of *Campylobacter jejuni* and *Campylobacter coli* strains, epidemiologic information, antimicrobial resistance genes detected, and multilocus sequence typing (MLST) results

	CCAMP1495	CCAMP1504	CCAMP1505
SRA accession no.^*a*^	SRR28886763	SRR28886762	SRR28886761
GenBank accession no.	GCA_036940345.1	GCA_036940375.1	GCA_036940365.1
Total reads before quality control	3,688,688	4,472,493	4,958,809
Total reads after quality control	1,776,630	1,781,306	1,755,452
Read length (bp)	150	150	150
Number of contigs	48	91	60
Genome size (bp)	1,800,000	1,800,000	1,700,000
G + C content (%)	30	31	31
Genes (total)	1,901	1,893	1,855
CDSs (total)	1,855	1,847	1,809
Genes (coding)	1,806	1,806	1,776
Genes (RNA)	46	46	46
Genome coverage (x)	623	755	847
N50 (kb)	122.7	329.9	146.2
Completeness (%)	99.48	98.27	98.49
Contamination (%)	0.21	0.89	0.71
City	Rio de Janeiro	Rio de Janeiro	Rio de Janeiro
Year	2013	2007	2007
Source	Human	Pig	Pig
Isolation type	Human—not specified	Animal—not specified	Animal—not specified
Species	*Campylobacter jejuni*	*Campylobacter coli*	*Campylobacter coli*
Genotype resistance	*gyrA*_T86I, *bla*_OXA-193_, *acr3*, and *arsP*	50S_L22_A103V, *acr3*, *tet*(O), *gyr*A_T86I, and 23S_A2075G	*gyrA*_T86I, *tet*(O), *acr3*, and 23S_A2075G
Plasmids length (bp)	−	3,303 and 2,421	3,304
Sequence Type (ST)	12332	12663	12333
Clonal Complex	21	828	−
Locus combination	*aspA* (2), *glnA* (1), *gltA* (5), *glyA,* (10), *pgm* (637), *tkt* (1), and *uncA* (5)	*aspA* (639), *glnA* (39), *gltA* (44), *glyA* (82), *pgm* (104), *tkt* (43), and *uncA* (36)	*aspA* (639), *glnA* (39), *gltA* (44), *glyA* (82), *pgm* (118), *tkt* (44), and *uncA* (36)

^*a*^ SRA, Sequence Read Archive.

## Data Availability

BioProject accession number: PRJNA986926. Accession numbers of assembled sequences: JAUHUC000000000, JAUHTZ000000000, and JAUHTY000000000. Biosample: SAMN35880995, SAMN35880998, and SAMN35880999.
